# The effects of a very low-energy ketogenic therapy on body composition, gut microbiota and metabolites in overweight subjects

**DOI:** 10.3389/fnut.2025.1714444

**Published:** 2026-01-09

**Authors:** Yeganeh Manon Khazrai, Claudia Di Rosa, Annamaria Altomare, Marta Giovanetti, Ludovica Di Francesco, Greta Lattanzi, Chiara Spiezia, Antonella Simone, Federica Coccaro, Giulia Costa, Michele Pier Luca Guarino, Silvia Manfrini

**Affiliations:** 1 Research Unit of Food Science and Human Nutrition, Department of Sciences and Technologies for Sustainable Development and One Health, Università Campus Bio-Medico di Roma, Rome, Italy; 2 Department of Sciences and Technologies for Sustainable Development and One Health, Università Campus Bio-Medico di Roma, Rome, Italy; 3 Operative Research Unit of Gastroenterology, Fondazione Policlinico Universitario Campus Bio-Medico, Rome, Italy; 4 Instituto René Rachou, Fundação Oswaldo Cruz, Belo Horizonte, Minas Gerais, Brazil; 5 Research Unit of Gastroenterology, Department of Medicine and Surgery, Università Campus Bio-Medico di Roma, Rome, Italy; 6 Unit of Endocrinology and Diabetes, Fondazione Policlinico Universitario Campus Bio-Medico, Rome, Italy

**Keywords:** gut metabolites, ketogenic diet, microbiota, obesity, VLEKT

## Abstract

Obesity is associated with chronic diseases and gut-brain axis disruptions, with diet influencing gut microbiota. This single-arm, uncontrolled study evaluated the effects of a 28 – day Very Low-Energy Ketogenic Therapy (VLEKT) on body weight, gut microbiota, and stool-derived metabolites in individuals with excess weight. Forty-one subjects underwent baseline (T0) assessment including anthropometry, bioimpedance analysis, gut microbiota profiling and completed FAST questionnaire before and after 28 days of VLEKT with meal replacements (T1). Follow-up was conducted at T1. Thirty-one participants completed the intervention (T1). Results showed significant reductions in body weight, BMI, and fat mass. Microbiota analysis revealed decreased Firmicutes and Actinobacteria, and increased Bacteroidetes and Verrucomicrobia. Genus-level changes included increases in *Bacteroides, Parabacteroides*, and *Akkermansia*, and decreases in *Streptococcus, Dorea, Blautia, Bifidobacterium*, and *Ruminococcus.* Stool metabolites showed decreased butyrate and lactate and increased propionate, vitamin K2, and GABA. Because the study lacked a control group and did not include systemic biomarkers or biochemical confirmation of ketosis, microbiota and metabolite changes cannot be linked to physiological effects. Overall, findings indicate short-term weight loss and stool microbiota modulation, but all microbiota-related outcomes remain exploratory due to the small sample size, short duration, and uncontrolled design.

## 1 Background

Obesity is a chronic, multifactorial disease characterized by an excessive and progressive accumulation of adipose tissue. According to the 2022 report from the World Health Organization (WHO), 59% of European adults are classified as overweight or with obesity (1).

Body Mass Index (BMI) is the principal criterion used by WHO guidelines to assess weight status relative to height. A BMI between 25.0 and 29.9 kg/m^2^ indicates overweight, while a BMI of 30 kg/m^2^ or higher defines obesity (2). Obesity is closely linked to chronic low-grade systemic inflammation, which contributes significantly to the development of various chronic diseases, including type 2 diabetes mellitus, metabolic syndrome, cardiovascular diseases and certain cancers. Additionally, obesity has been linked to alterations in gut microbiota composition, a condition known as dysbiosis, which contributes to the persistence of low-grade inflammation (3–5).

The human body hosts approximately 3.8 × 10^13^ microorganisms, primarily residing in the gastrointestinal tract (2). These microorganisms colonize the intestinal mucosa and lumen shortly after birth and are influenced by factors such as delivery mode (vaginal vs. cesarean), early nutrition (breastfeeding vs. formula feeding) and host genotype. Each individual harbors 150 to 400 microbial species, with a predominance of *Bacteroidetes*, *Firmicutes*, *Actinobacteria,* and *Proteobacteria* (6). Importantly, gut microbiota composition can change over time within the same individual, reflecting diet, lifestyle, and physiological rhythms. This temporal variability accompanies inter-individual differences that persist throughout life, from early development to older age (6). Dysbiosis, an imbalance in microbial populations, has been linked to altered intestinal motility, low grade-inflammation, enteric nervous system dysfunction, and vagal nerve alterations, all of which may contribute to IBS symptoms such as abdominal pain, bloating, and irregular bowel movements (7–10). In this study, IBS symptoms are defined according to the Rome IV criteria which describe recurrent abdominal pain associated with defecation and/or changes in stool frequency or form (11).

Under normal physiological conditions, the gut microbiota plays several beneficial roles including: (i) metabolism of carbohydrates and lipids; (ii) synthesis of vitamins and amino acids; (iii) epithelial cell proliferation; (iv) protection against pathogen; (v) and modulation of hormone production (2). Diet plays a central role in shaping microbial composition, influencing bacterial growth and the production of key metabolites such as short-chain fatty acids (SCFAs) and other bioactive compounds, which have significant implications for host health (12).

Gut microbiota composition is influenced by diet, medications, gut mucosal integrity, immune function, and the microbiota itself. Among these factors, diet exerts the most profound effect.

Recent studies highlight the influence of different dietary patterns on gut microbiota composition. In particular, diets rich in animal proteins and fats are associated with increased proportion of *Bacteroidetes* and *Firmicutes*, while vegetarian diets are linked to higher levels of *Prevotella* (13). These associations highlight how nutritional habits can shape both the structure and the metabolic functions of the gut microbiota (14). Recent research has explored the use of Very Low - Energy Ketogenic Therapy (VLEKT) (formerly known as Very Low Calorie Ketogenic Diet, VLCKD) as a promising dietary strategy to fight obesity and its comorbidities. This protocol involves a hypocaloric (<800 kcal/day), low-carbohydrate (<50 g of carbohydrates/day) normoproteic (0.8–1.5 g /kg desirable body weight/day) diet. In addition to weight management, VLEKT has been shown anti-inflammatory effects by modulating lipid metabolism, reducing oxidative stress and lowering inflammatory markers (15, 16).

However, the effects of hypocaloric ketogenic interventions on the gut microbiota remain incompletely understood. Short-term dietary restriction alone may drive substantial microbial shifts, independent of macronutrient ratios. Furthermore, the absence of a control group, makes it impossible to distinguish VLEKT-related changes from normal temporal fluctuations of the gut microbiota.

The present study aimed to evaluate the impact of a 28-day VLEKT protocol on body weight and composition, gut microbiota composition, and microbial metabolite production in individuals with excess weight. Given the exploratory single-arm design, these findings should be interpreted as descriptive and hypothesis-generating rather than causal.

## 2 Materials and methods

### 2.1 Study participants

Forty one (*n* = 41) subjects with excess weight were consecutively enrolled at the Endocrinology and Gastroenterology Units of Campus Bio-Medico University Hospital in Rome, based on predefined inclusion and exclusion criteria. Each participant underwent a clinical evaluation prior to enrollment to assess eligibility for a ketogenic dietary protocol.

Inclusion criteria:

- Age > 18 years
- Metabolically healthy overweight individuals (MHOW) (BMI ≥ 25–29.9 kg/m^2^) Metabolically healthy individuals with obesity (MHO) (BMI ≥ 30 kg/m^2^) (Metabolically healthy are based on the absence of: hypertension, dyslipidemia, impaired fasting glucose, insulin resistance or type 2 diabetes, metabolic syndrome, NAFLD or other metabolic liver diseases, chronic systemic inflammation)
- Subjects deemed suitable for a ketogenic diet by an endocrinologist, in accordance with the 2019 Società Italiana di Endocrinologia (SIE) guidelines (15)
- Written informed consent obtained for participation in the study protocol

Exclusion criteria:

- Subjects already enrolled in other clinical studies
- Pregnancy or lactation
- Kidney or liver insufficiency
- Subjects diagnosed with type 1 diabetes, latent autoimmune diabetes in adults (LADA)
- Presence of any metabolic comorbidity including: type 2 diabetes, hypertension, dyslipidemia, metabolic syndrome, NAFLD
- Obstructive sleep apnea syndrome (OSAS) or osteoarticular disorders
- Subjects with unstable angina, recent stroke, heart failure (<12 months) or cardiac arrhythmias
- Use of systemic antibiotics, gastrointestinal acting medications, prebiotics, or probiotics within the previous month
- Presence of organic diseases (inflammatory, neoplastic, metabolic disorders affecting gastrointestinal motility)
- Presence of psychiatric disorders including anorexia nervosa, bulimia nervosa, obsessive-compulsive disorder, major depressive disorder or dysthymia.

Subjects with type 1 diabetes, recent cardiovascular events, or heart failure were excluded because these conditions represent contraindications to ketogenic therapy according to the SIE guidelines, and to avoid potential metabolic complications (e.g., ketoacidosis) or risks associated with rapid weight loss.

The study was conducted in accordance with the Declaration of Helsinki and approved by the Ethics Committee Campus Bio – Medico University of Rome (Approval No. 47.22 dated 19/07/2022).

### 2.2 Study design

The present study was designed as a single-arm, uncontrolled pre–post interventional study, in which each participant served as his or her own control. This design was chosen to allow within-subject comparisons before and after the dietary intervention in the absence of a control group. Therefore, the results should be interpreted as exploratory and descriptive, without causal inference. During the enrollment phase (T0–7) participants were consecutively enrolled according to inclusion and exclusion criteria and signed the written informed consent before being included in the study.

All enrolled participants followed the 28-day VLEKT regimen after a 7-day period of habitual diet. During the baseline period (from T0-7 to T0), participants maintained their usual real-world eating patterns, with no structured dietary plan or professional nutritional guidance. The habitual diet phase was not standardized, as participants were instructed to maintain their spontaneous eating habits to reflect real-life conditions.

Fecal samples for metagenomic analysis were collected at the end of the first week (baseline) and again after 28-day dietary intervention. Participants were provided with written instructions and educational materials describing the dietary protocol and had access to study staff for clarifications if needed. Compliance with the diet was monitored through the completion of a daily food diary, in which participants recorded all foods consumed as well as any side effects experienced. Additional adherence and symptom monitoring was performed through completion of the FAST questionnaire at both time points. All participants received the same brand and formulation of meal replacements to ensure consistency of the ketogenic intervention across individuals.

**Figure 1:**
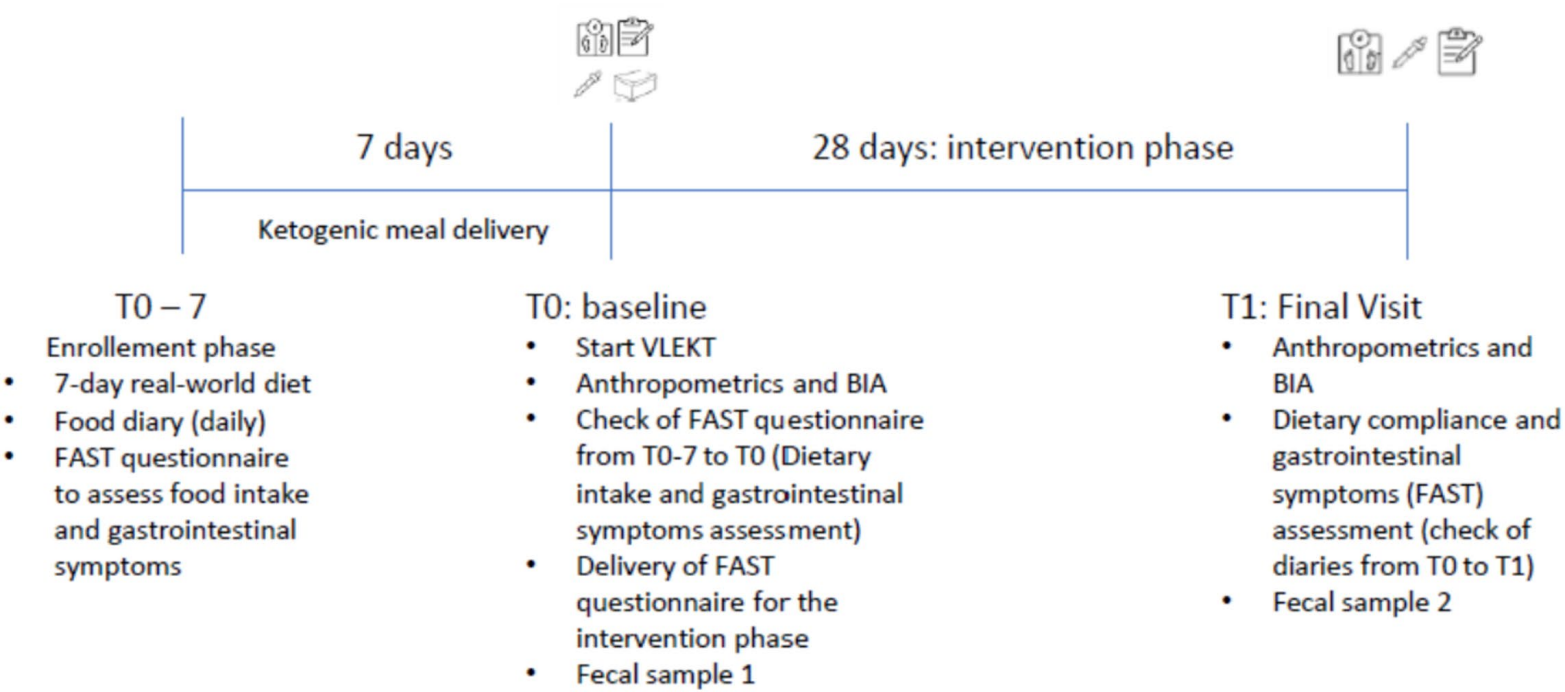
Study design.

At the end of the 28-day VLEKT protocol, participants collected a second fecal sample using a provided kit and again underwent anthropometric data measurements and body composition analysis using vector bioimpedance analysis (BIA) (Figure 1).

Anthropometric and body composition assessments, were conducted both at the beginning (T0) and at the end (T1) of the study, allowing for the quantification of body changes induced by the VLEKT protocol. These evaluations were performed by trained personnel following standardized procedures to ensure measurement consistency. Upon completion of the study, participants were provided with personalized dietary protocols and two fecal sampling kits, along with detailed instructions for their use. The post-intervention dietary recommendations were delivered only after the T1 assessments to avoid influencing the intervention period.

#### 2.2.1 Anthropometric parameters measurement

Participants underwent an evaluation of anthropometric parameters, which included the measurement of body weight using a Seca 761 mechanical scale, and height using a Seca 213 mobile stadiometer. Waist, and hip circumferences were measured using a SECA 201 non-elastic measuring tape. All measurements were performed in light clothing, without shoes, and following standardized procedures to minimize inter-operator variability. Body weight and circumferences were assessed at the same time of day during both visits to reduce diurnal fluctuations.

#### 2.2.2 Body composition evaluation

Body composition was assessed using the BIA 101 BIVA PRO impedance analyzer (Akern), which enables the evaluation of nutritional, muscular and hydro-electrolytic status. The analysis provided values for total body water (TBW, %), extracellular water (ECW, %), and intracellular water (ICW, %). Additionally, it allowed for the quantification of lean body mass (fat-free mass, FFM, %), fat mass (FM, %), metabolically active cell mass (body cell mass, BCM, %), and phase angle (PhA). Measurements were performed under standardized conditions (after an overnight fast, after voiding, and avoiding strenuous physical activity in the previous 12 h) to minimize hydration-related variability. All assessments were carried out by trained operators following manufacturer guidelines.

#### 2.2.3 Nutritional intervention protocol

The nutritional intervention was based on a Very Low Energy Ketogenic Therapy (VLEKT) with meal replacements from the same brand, lasting 28 days. The dietary protocol was characterized by a total energy intake of less than 800 kcal/day, with carbohydrate intake restricted to less than 50 g/day to induce and maintain a state of nutritional ketosis. Protein intake was individualized according to each participant’s desirable body weight (0.8–1.5 g/kg body weight/day). Lipid intake was limited to 20–30 g/day for both men and women. To ensure methodological consistency, all participants received standardized instructions regarding hydration, permitted foods, and management of possible side effects. To prevent the risk of nutritional deficiencies, all participants received supplementation with sodium and potassium bicarbonate (1.5–1.2 g/day), a standard multivitamin and omega-3 fatty acids (1 g/day). All meal replacements were produced by the same manufacturer and provided in standardized portion sizes to ensure uniformity of macronutrient intake across participants.

The dietary plan included:

- for women: three main meals and one snack per day, consisting of two powdered meal replacements (at breakfast and dinner) and two solid meal replacements (at lunch and snack).
- For men: three main meals and two snacks per day, consisting of three powdered meal replacements (at breakfast, morning snack, and dinner) and two solid meal replacements (at lunch and afternoon snack).

The liquid meal replacements were powdered products, such as smoothies, chocolate drinks, milkshakes, and creamy soups, which required reconstitution with water. The solid meal replacements included ready-to-eat savory snacks, wafers, and bakery items. These products contained a standardized mixture of functional fibers (including inulin, resistant starch, acacia fiber, bamboo fiber, guar gum, and psyllium), which contributed to the overall dietary fiber intake during the intervention.

The nutritional composition of the powdered liquid meal replacements (considered per portion) was as follows:

- Breakfast (portion 25 g): ~ 93 kcal, 0.9 g fat, 18 g protein, 2.5 g carbohydrate, 1.4 g fiber
- Lunch and Dinner: (portion 25 g) ~ 91 kcal, 0.9 g fat, 18 g protein, 2.4 g carbohydrate, 0.8 g fiber
- Snack (portion 25 g): ~94 kcal, 0.7 g fat, 18 g protein, 3.5 g carbohydrate, 1.2 g fiber

The ready-to-eat solid snacks included:

- Savory snacks (portion 30 g): ~117 kcal, 3.7 g fat, 14 g protein, 4.9 g carbohydrate, 5 g fiber
- Savory snacks (portion 50 g): ~184 kcal, 8 g fat, 15 g protein, 4.2 g carbohydrate, 20 g fiber
- Wafers (portion 35 g): ~169 kcal, 9 g fat, 15 g protein, 5 g carbohydrate, 2.6 g fiber

The ready-to-eat solid meals provided for lunch or dinner were:

- Pasta (portion 50 g): ~151 kcal, 0.8 g fat, 20 g protein, 6 g carbohydrate, 20 g fiber
- Substitute for bread (portion 50 g): ~178 kcal, 7.8 g fat, 14 g protein, 4.6 g carbohydrate, 20 g fiber
- Bread (portion 70 g): ~183 kcal, 8.8 g fat, 15 g protein, 6.3 g carbohydrate, 7.5 g fiber

Participants were instructed to record all food intake in daily food diaries, which were used to monitor adherence to the ketogenic protocol.

#### 2.2.4 Food and symptoms times (FAST) questionnaire

All participants were instructed to complete the FAST Questionnaire (17) which collects detailed information on dietary intake and any gastrointestinal symptoms such as abdominal pain, bloating, meteorism, frequency, urgency of defecation, stool forms and Bristol Stool Scale (BSS). This validated self-reported tool was used to monitor both dietary adherence and gastrointestinal tolerability throughout the intervention. This questionnaire was used as an additional tool to monitor adherence and tolerability to the intervention.

##### 2.2.4.1 Dietary intake

Participants were asked to keep a food diary and report the following information:

- Meal type (e.g., breakfast, snack, dinner),
- Time of consumption,
- Location of the meal (e.g., home, restaurant),
- Food consumed (including type and quantity)
- Method of preparation or cooking.

Daily food diaries were reviewed to evaluate adherence to the prescribed macronutrient targets and to identify any deviations from the ketogenic protocol.

##### 2.2.4.2 Gastrointestinal symptoms

The gastrointestinal symptoms investigated were abdominal pain, bloating/abdominal distension, meteorism, stool frequency, urgency of defecation, straining during defecation, and pre-defecation abdominal pain.

For abdominal pain, bloating, and meteorism, symptom frequency was quantified using the FAST time-slot method. The 24-h period was divided into three 8-h time slots (00:00–08:00, 08:00–16:00, 16:00–24:00) to allow for a simple and standardized recording of symptom occurrence throughout the day. Each symptom was assigned a score based on the number of time slots in which it was reported:

- 1 point = symptom reported in one time slot
- 2 points = symptom reported in two time slots
- 3 points = symptom reported in all three time slot.

A score of 0 was assigned when no symptom occurred.

Stool frequency was recorded as the exact number of bowel movements per day.

Straining, pre-defecation abdominal pain, and urgency of defecation were assessed using a 5-point Likert scale, where higher scores indicated greater severity of the symptom.

This combined scoring approach allowed for a detailed and differentiated quantification of gastrointestinal symptoms throughout the intervention.

##### 2.2.4.3 Bristol stool scales (BSS)

The Bristol Stool Scale is a useful tool to evaluate the prevalent stool type and consistency, that ranges from hard lumps to watery stools. The shape and consistency of stools depend on the time they spend in the colon. Stool types 1 and 2 define a higher or lower degree of constipation due to excessive retention in the intestine and difficulty in expulsion. Type 3 is similar to an ideal condition that implies a need for the body to consume more fiber and/or water. Type 4 represents the ideal condition, with easily expelled stools followed by the feeling of complete emptying; type 5 is also considered acceptable. Types 6 and 7 are associated with a different degree of diarrhoea (18). The BSS classification was used to categorize stool consistency at both T0 and T1 to allow a standardized evaluation of bowel habit changes.

#### 2.2.5 Fecal sample kit Wellmicro®

To characterize gut microbiota composition, all participants were provided with a Wellmicro® self-collection kit for fecal sample collection. Samples were obtained at two time points: at baseline (following 7 days of habitual diet) and at the end of the 28-day VLEKT intervention.

Microbiome analysis was conducted using the Wellmicro® standardized metagenomic workflow. DNA extraction was performed using a proprietary high-yield protocol. Sequencing was carried out using the Illumina® MiSeq platform, with an average sequencing depth of approximately 75,000–100,000 reads per sample.

Raw sequencing data underwent adaptor trimming, quality filtering, and chimera removal. Taxonomic classification was performed using QIIME2 (version 2023.5) with reference alignment to the SILVA rRNA database (v138). Relative abundance was calculated using total-sum scaling (TSS) for normalization.

Microbial diversity was assessed using *α*-diversity metrics (Shannon index and observed species) and *β*-diversity (Bray–Curtis dissimilarity, evaluated qualitatively).

The Wellmicro® report also provided a Dysbiosis Index, calculated via multivariate comparison of individual microbiota profiles to a reference database of healthy adults. Values close to zero indicate eubiosis, whereas progressively positive or negative values denote increasing dysbiosis.

Metabolite profiling included: acetate, butyrate, propionate, succinate, lactate, GABA, histamine, indole, indoleacetic acid, indolepropionic acid, tryptamine, serotonin, trimethylamine (TMA), polyphenols, B-group vitamins, vitamin K₂, gluten degradation products, mucolysis, proteolysis, lipopolysaccharides (LPS), secondary bile acids, ethanol, hydrogen sulfide, and methane.

- *Metabolite scoring:* −1 to +1 = optimal; values outside this range = suboptimal.

Functional indices evaluated included: immune homeostasis, mucosal homeostasis, glucose metabolism, lipid metabolism, pro-inflammatory activity, antimicrobial activity, gut–brain axis, gut–liver axis, gut–skin axis, and circadian rhythm.

- *Functional scoring*: 0–3 = high alteration; 4–7 = moderate alteration; >8 = optimal.

### 2.3 Statistical analysis

Anthropometric parameters, body composition variables, and dietary intake data were analyzed using GraphPad Prism (version 10.2.2). Data distribution was assessed using the Shapiro–Wilk test. When normality was confirmed (*p* > 0.05), paired t-tests were performed; otherwise, the Wilcoxon signed-rank test was used. Results were expressed as mean ± SD for normally distributed variables and median (interquartile range) for non-normal or ordinal data. Bristol Stool Scale classifications were analyzed using a contingency test. Statistical significance was set at *p* < 0.05.

Microbiota, microbial metabolite, and functional index data were analyzed using RStudio (version 2024.02.0 + 492). Normality was assessed using the Shapiro–Wilk test. Paired t-tests were applied when normality assumptions were met, while the Wilcoxon signed-rank test was used otherwise. Given the limited sample size and the exploratory nature of this pilot study, no corrections for multiple comparisons (e.g., FDR) were applied, as these procedures would disproportionately increase the risk of Type II error and potentially obscure biologically meaningful patterns. Accordingly, results were interpreted within an exploratory analytical framework. Microbial relative abundances were computed after normalization of sequencing reads, and alpha- and beta-diversity metrics were calculated using standard ecological indices.

## 3 Results

### 3.1 Study population

**Figure 2:**
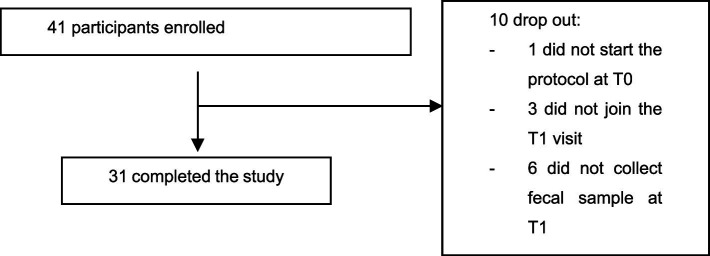
Participant flow chart.

Out of 41 enrolled participants, 31 completed the study (Figure 2). At baseline, the 31 participants had a mean age of 48.0 ± 10.54 years and a mean height of 1.60 ± 0.07 m. These 31 individuals constitute the “whole population” referred to throughout the manuscript. Fourteen of them also reported a diagnosis of IBS; this subgroup was analyzed separately in an exploratory manner, without comparative statistical testing against non-IBS participants due to limited sample size. This approach ensured that subgroup findings remained descriptive and appropriately interpreted.

### 3.2 Anthropometrical parameters and body composition

At T1, following the VLEKT intervention, a significant reduction in body weight (*p* < 0.0001), BMI (*p* < 0.0001), waist circumference (WC) (*p* < 0.0001) and hip circumference (*p* < 0.0001) was observed. Moreover, a significant decrease in fat mass and a significant increase in fat-free mass were observed from T0 to T1 (*p* = 0.0005 for both). It should be noted that the increase in fat-free mass refers to the *percentage* of FFM, whereas absolute FFM (kg) showed a slight reduction due to overall weight loss; this indicates that fat decreased proportionally more than lean tissue. Phase angle, total body water (TBW), extra cellular water (ECW), intra cellular water (ICW) and body cell mass (BCM) did not show significant changes from T0 to T1 (Table 1). These findings suggest improved body composition primarily driven by caloric restriction, while indices of cellular integrity and hydration remained stable.

**Table 1:** Significant and not significant variations in anthropometric and body composition parameters in the total whole population.

Anthropometric and body composition parameters	T0	T1	*p*-value
Body weight (kg) (mean ± SD)	79.5 ± 14.1	74.3 ± 13.6	<0.0001
BMI (kg/m^2^) (mean ± SD)	30.9 ± 4.9	28.9 ± 4.8	<0.0001
Waist circumference (cm) (mean ± SD)	91.2 ± 12.8	84.3 ± 11.3	<0.0001
Hip circumference (cm) (mean ± SD)	110.3 ± 9.8	105.8 ± 10.1	<0.0001
Fat-free mass (FFM) (%)	62.8 ± 6.1	64.6 ± 6.5	<0.05
Fat Mass (FM) (%)	37.2 ± 6.1	35.4 ± 6.5	<0.05
Total body water (TBW) (%)	46.0 ± 4.5	45.6 ± 8.0	0.6961 (n.s)
Extra Cellular water (ECW) (%)	47.2 ± 2.8	45.5 ± 6.8	0.1276 (n.s)
Intra Cellular water (ICW) (%)	52.8 ± 2.8	53.1 ± 3.2	0.4854 (n.s)
Body Cell Mass (BCM) (%)	52.1 ± 3.0	52.5 ± 3.4	0.4720 (n.s)
Phase angle	5.7 ± 0.6	5.8 ± 0.7	0.4369 (n.s)

Values are expressed as mean ± SD. Statistical test: *t*-test.

### 3.3 Food diaries

As expected, a significant reduction in carbohydrate intake was observed (*p* < 0.0001) consistent with the VLEKT protocol, which provides less than 50 g of carbohydrates per day. Lipid intake also significantly decreased (*p* < 0.0001), while dietary fiber intake showed a significant increase (p < 0.0001). This rise in total fiber intake reflects the fiber content of the standardized meal-replacement products used during the intervention, which contain added functional fibers (e.g., inulin, resistant starch, acacia fiber, psyllium), rather than an increase in whole-food fiber consumption. In contrast, the daily intake of protein (in grams) did not change significantly (Table 2). This stability in protein intake is consistent with the design of the VLEKT protocol, in which protein amounts are individualized and maintained throughout the intervention. Because dietary data were self-reported, the observed changes—particularly for fiber—should be interpreted with caution due to potential reporting inaccuracies.

**Table 2:** Dietary intake in the whole population.

Dietary intake variables	T0	T1	*P*-value
Carbohydrates (g)	160.0 ± 51.0	21.0 ± 0.0	<0.0001
Proteins (g)	69.0 ± 19.0	71.0 ± 4.0	0.5415 (n.s.)
Lipids (g)	72.0 ± 22.0	35.0 ± 1.0	<0.0001
Dietary fibers (g)	13.0 ± 4.0	39.0 ± 0.0	<0.0001

Values are expressed as mea ± SD. Statistical test: *t*-test.

### 3.4 Gastrointestinal symptoms

**Figure 3:**
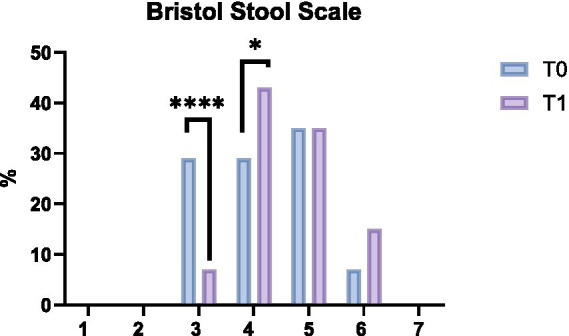
Bristol Stool scale in the IBS subgroup. Values are expressed as median. Statistical test: contingency test. *p < 0.05; ****p < 0.0001.

Gastrointestinal symptoms were also assessed using the FAST questionnaire. This tool was administered to the whole population (*n* = 31) and allowed the identification of 14 subjects affected by IBS who were subsequently subjected to a separate subgroup analysis. Given the small sample size, this subgroup analysis should be considered exploratory and descriptive rather than confirmatory. In these 14 subjects, a significant reduction in abdominal bloating (*p* < 0.05) was observed following the ketogenic protocol. No significant changes were found for meteorism, straining, pre-evacuation pain and urgency, although a moderate, non-significant reduction in abdominal pain was observed. At baseline (T0), according to the Bristol Stool Scale, 35.7% of IBS participants reported a score of 5, 28.6% a score of 3, and another 28.6% a score of 4, while only 7% reported a score of 6. At T1, 42.9% of participants reached a score of 4 (*p* < 0.05), and 7.1% reported a score of 3 (*p* < 0.0001). No significant changes were observed for scores 5 and 6 (Figure 3). Because stool types 3 and 4 fall within the normal range of consistency, the shift toward type 4 should be interpreted as a normalization of bowel habits rather than a clinically relevant pathological change.

Evacuation frequency did not significantly change, either in the whole population or in the IBS subgroup. In fact, in the whole population, it was 8.0 ± 4.2/week at T0 and 7.3 ± 3.1/week after the VLEKT, while in the IBS subgroup, it was 8.1 ± 4.3/week at T0 and 8.1 ± 3.3/week at T1. These findings indicate that stool frequency remained stable during the intervention, suggesting no substantial effect on bowel transit. Given the small number of IBS participants, these results should be interpreted as descriptive rather than clinically meaningful.

### 3.5 Fecal microbiota analysis

#### 3.5.1 Microbiota composition

**Figure 4:**
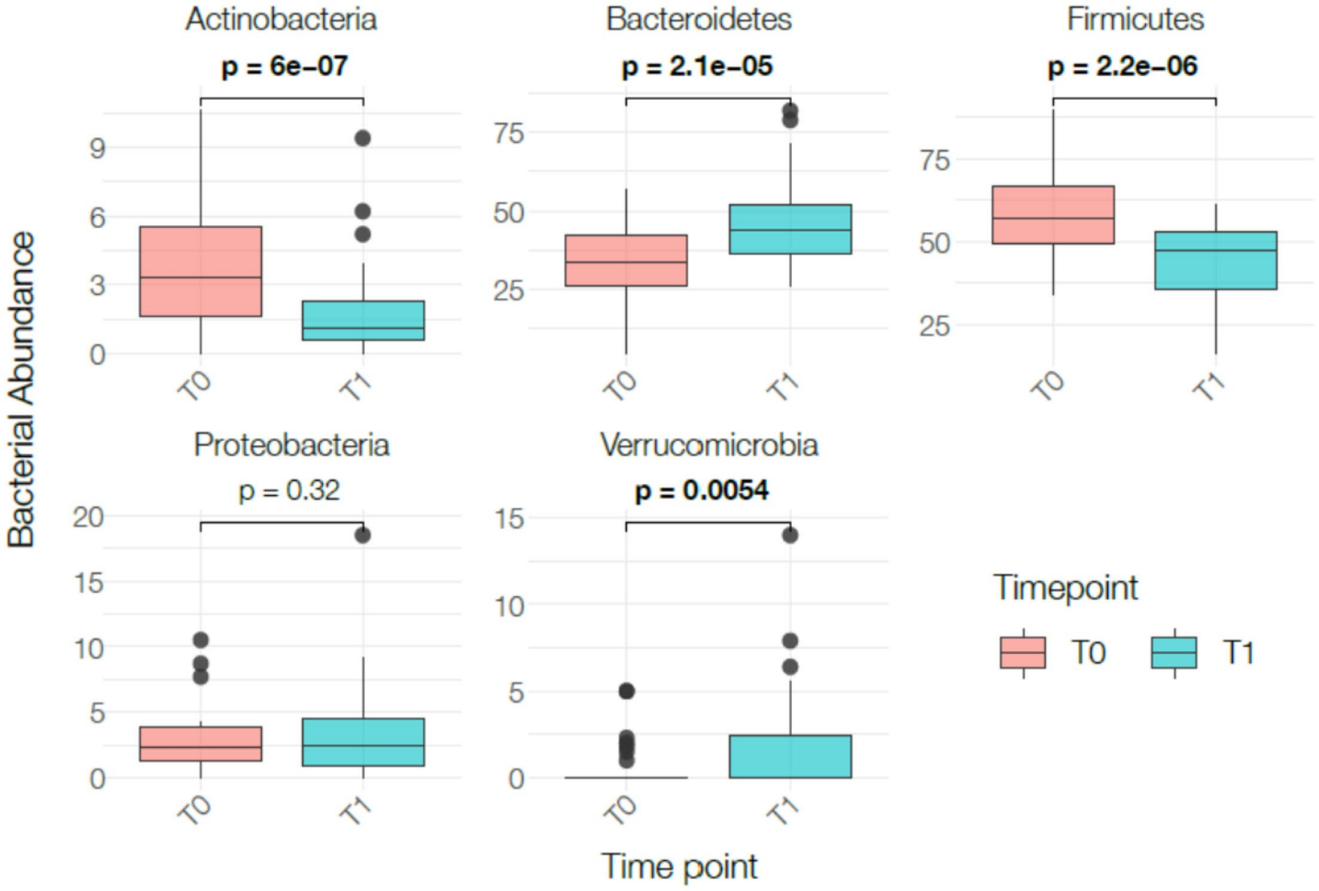
Changes in bacterial phylum-level relative abundance between baseline (T0) and post-intervention (T1). Box plots display the distribution of normalized relative abundance (total-sum scaling) for Actinobacteria, Bacteroidetes, Firmicutes, Proteobacteria, and Verrucomicrobia at both time points. Boxes represent the interquartile range (IQR), horizontal lines indicate median values, whiskers show the full range, and individual data points are overlaid. Statistical comparisons were performed using paired *t*-test or Wilcoxon signed-rank test according to distribution. Statistically significant differences are indicated using bold *p*-values.

The number of species significantly increased (*p* < 0.05) while the *Firmicutes/Bacteroidetes* ratio significantly decreased (*p* < 0.05). Dysbiosis and biodiversity indexes did not show significant changes. These indexes represent stool-derived computational estimates and should not be interpreted as clinical measures of intestinal function. After 1 month of VLEKT *Actinobacteria* (*p* < 0.0001) and *Firmicutes* (p < 0.0001) significantly decreased, while *Bacteroidetes* (*p* < 0.0001) and *Verrucomicrobia* (*p* < 0.05) significantly increased. These shifts are commonly observed during hypocaloric diets and may reflect changes in nutrient availability rather than macronutrient-specific effects. Moreover, in the absence of a control group, it is not possible to distinguish intervention-related changes from normal temporal fluctuations in microbiota composition. No significant changes were observed for *Proteobacteria* (Figure 4).

**Figure 5:**
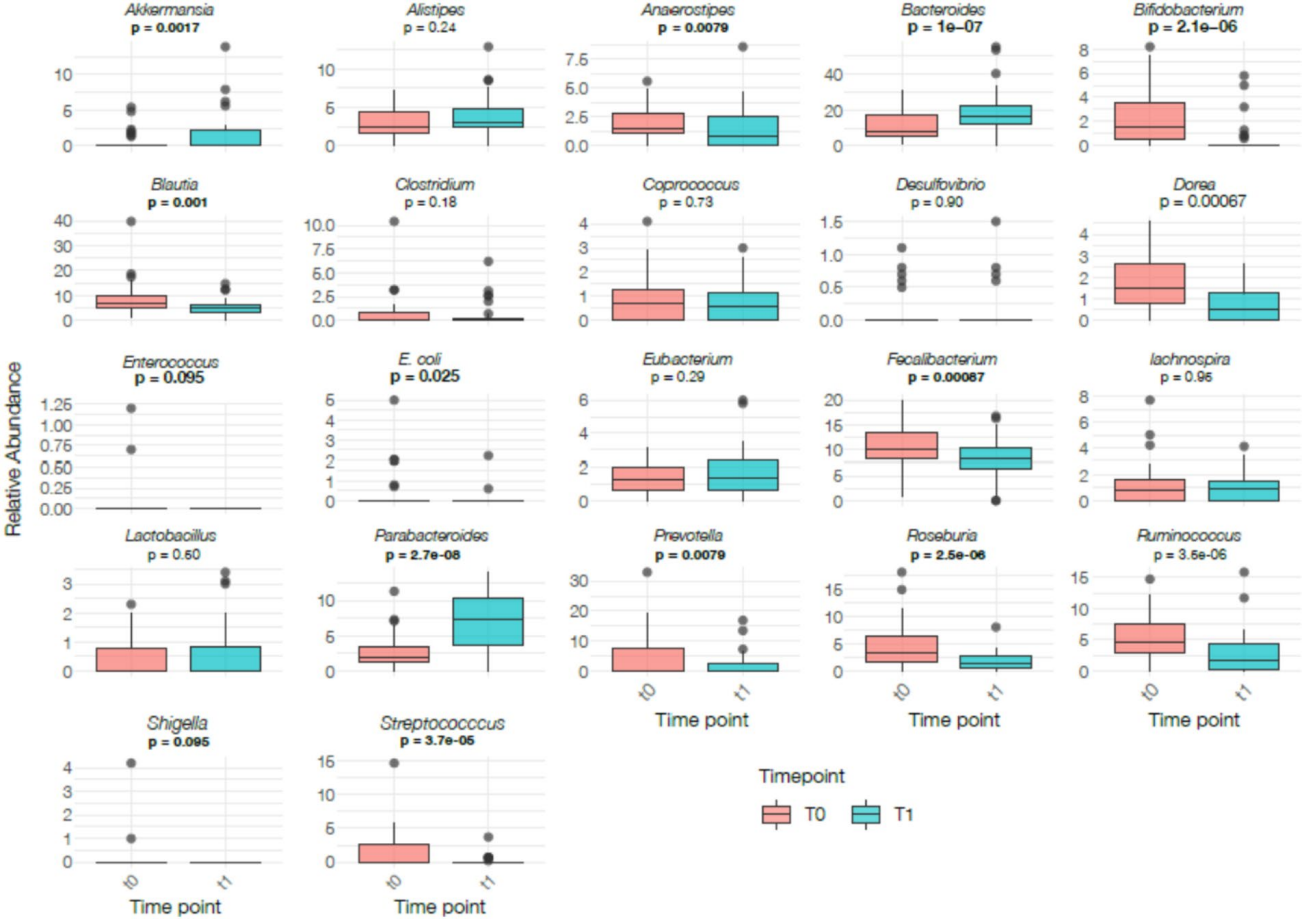
Genus-level bacterial relative abundance at baseline (T0) and post-intervention (T1). Each panel displays box plots representing the distribution of relative abundance for a specific bacterial genus across participants at both time points. Boxes denote the interquartile range (IQR), horizontal lines indicate the median, and whiskers represent the range. Individual data points are overlaid. Statistical comparisons were performed using paired *t*-test or Wilcoxon signed-rank test according to data distribution. Statistically significant differences are indicated by bold *p*-values. Relative abundance is expressed as normalized proportions (total-sum scaling).

The relative abundance of *Akkermansia* (*p* < 0.05), *Bacteroides* (*p* < 0.0001), and *Parabacteroides* (*p* < 0.0001) significantly increased. Conversely, the abundance of several genera significantly decreased, including *Anaerostipes* (*p* < 0.05), *Bifidobacterium* (*p* < 0.0001), *Blautia* (*p* = 0.001), *Dorea* (*p* < 0.05), *E. coli* (*p* < 0.05), *Faecalibacterium* (*p* < 0.05), *Prevotella* (*p* < 0.05), *Roseburia* (*p* < 0.0001), *Ruminococcus* (*p* < 0.0001), and *Streptococcus* (*p* < 0.0001). Additionally, *Enterococcus* and *Shigella* were completely absent at T1 (p < 0.05 in both cases). No significant changes were observed for *Alistipes*, *Clostridium, Coprococcus, Desulfovibrio, Eubacterium, Lachnospira*, and *Lactobacillus* (Figure 5). These variations should be interpreted with caution, particularly the reductions in genera generally considered beneficial (e.g., *Bifidobacterium*, *Blautia*, *Faecalibacterium*, *Roseburia*). This pattern may reflect reduced fermentable substrates under caloric restriction rather than direct effects of the ketogenic macronutrient profile. In addition, without a control group, genus-level fluctuations cannot be distinguished from normal temporal variability in gut microbiota composition.

#### 3.5.2 Metabolic and functional indexes

Regarding metabolic indexes, a significant reduction was observed in the production of butyrate (*p* < 0.0001), lactate (*p* < 0.0001), indolepropionic acid (*p* < 0.0001), and tryptamine (*p* < 0.05). Conversely, the levels of GABA (*p* < 0.0001), indole (*p* < 0.0001), propionate (*p* < 0.0001), serotonin (*p* = 0.023), trimethylamine (*p* < 0.05), vitamin K2 (*p* < 0.0001), and proteolysis (*p* < 0.05) significantly increased. No significant changes were observed in other metabolic indexes. These metabolite values arise exclusively from stool analysis and reflect microbial metabolic output rather than systemic biochemical concentrations; therefore, their physiological interpretation remains exploratory.

**Figure 6:**
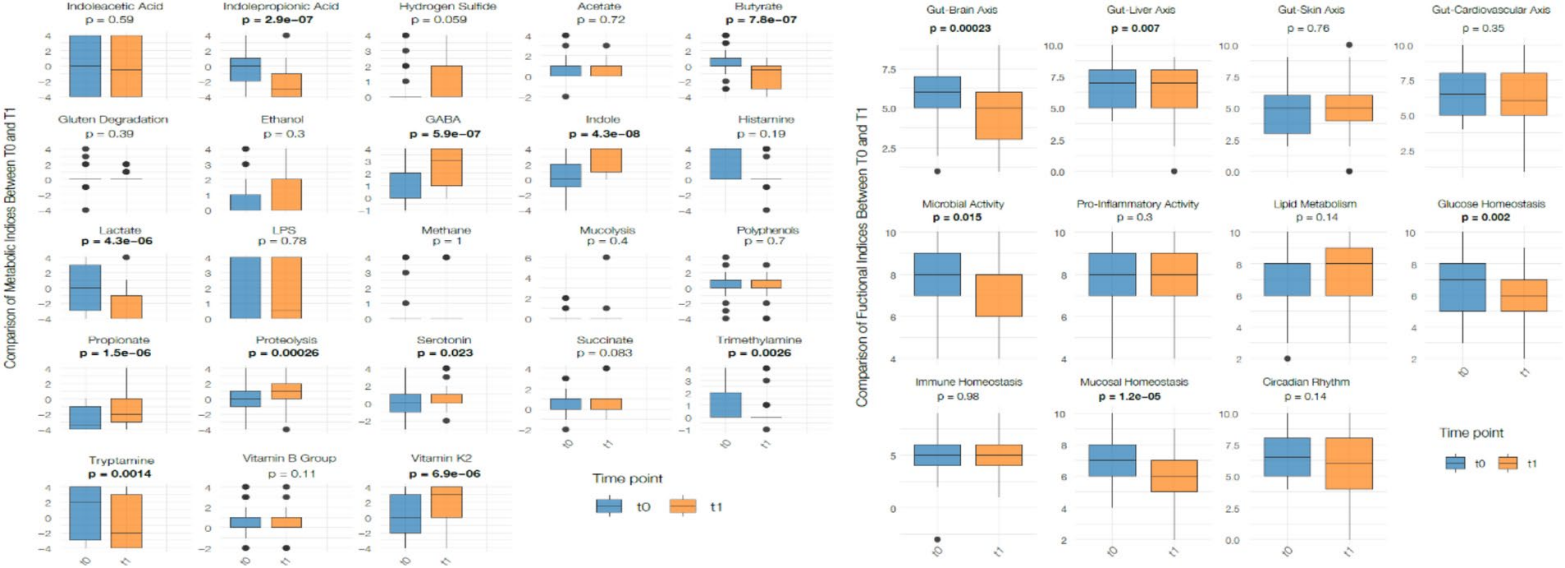
Changes in metabolic (left panel) and functional (right panel) indices between baseline (T0) and post intervention (T1) in the whole study population. Box plots display normalized values (total-sum scaling) and show individual distribution, median, interquartile range (IQR) and whiskers. Statistical comparisons were conducted using paired *t*-test or Wilcoxon signed-rank test depending on data distribution. Significant differences are indicated with bold *p*-values.

Regarding functional indexes, a significant reduction was observed in both gut-brain axis (*p* < 0.05) and liver-gut axis scores (*p* < 0.05). Additionally, mucosal homeostasis (*p* < 0.0001) and glucose homeostasis (*p* < 0.05) scores significantly decreased. A significant reduction was also observed in microbial activity (*p* < 0.05). No significant changes were detected in other functional indexes (Figure 6). These scores are computationally inferred from taxonomic profiles and are not validated clinical biomarkers. As such, they should not be interpreted as direct measurements of host neuroendocrine, hepatic, immune, or metabolic function. Given the absence of systemic physiological markers and the small sample size, these findings remain descriptive and exploratory.

#### 3.5.3 IBS subgroup

**Figure 7:**
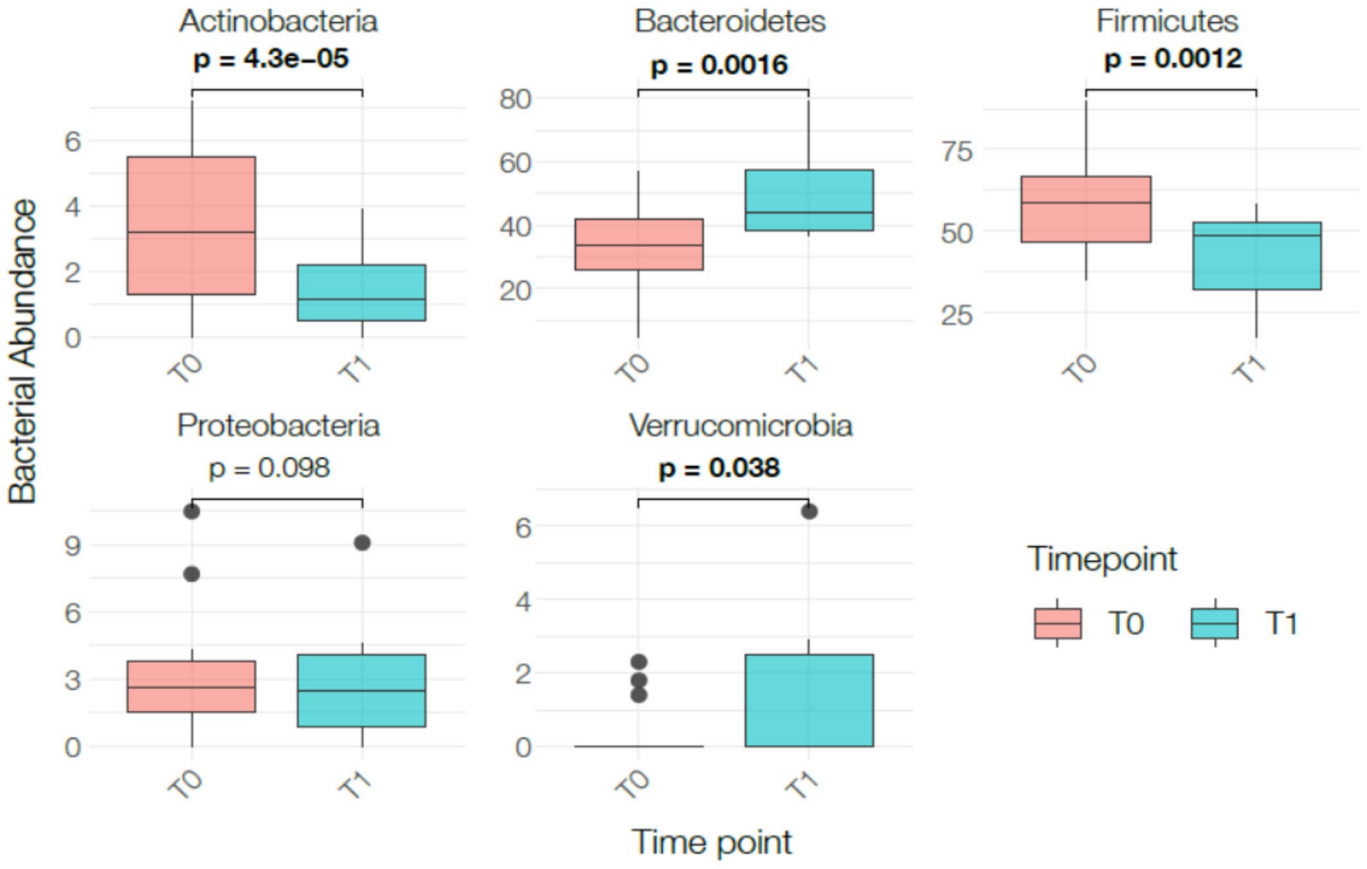
Changes in bacterial phylum-level relative abundance between baseline (T0) and post-intervention (T1) in the IBS subgroup. Box plots represent normalized relative abundance (total-sum scaling) for Actinobacteria, Bacteroidetes, Firmicutes, Proteobacteria and Verrucomicrobia. Boxes illustrate the IQR, median is shown by the central line, and whiskers indicate the full range. Statistical comparisons were conducted using paired t-test or Wilcoxon signed-rank test depending on distribution. Statistically significant differences are highlighted with bold *p*-values.

The number of species increased also in the subgroup with IBS (*p* < 0.05) with a significant reduction in *Firmicutes/Bacteroidetes* ratio (*p* < 0.05). Even in the IBS subgroup Actinobacteria (*p* < 0.0001) and Firmicutes (*p* < 0.05) decreased while Bacteroidetes (*p* < 0.05) and Verrucomicrobia (*p* < 0.05) increased. No changes in Proteobacteria were observed (Figure 7). Given the limited number of IBS participants (*n* = 14), these results should be interpreted as exploratory and descriptive only. The observed phylum-level shifts are consistent with those found in the whole population and may reflect general responses to caloric restriction rather than IBS-specific patterns. Furthermore, without a control group, these changes cannot be distinguished from normal temporal variability of the gut microbiota.

**Figure 8:**
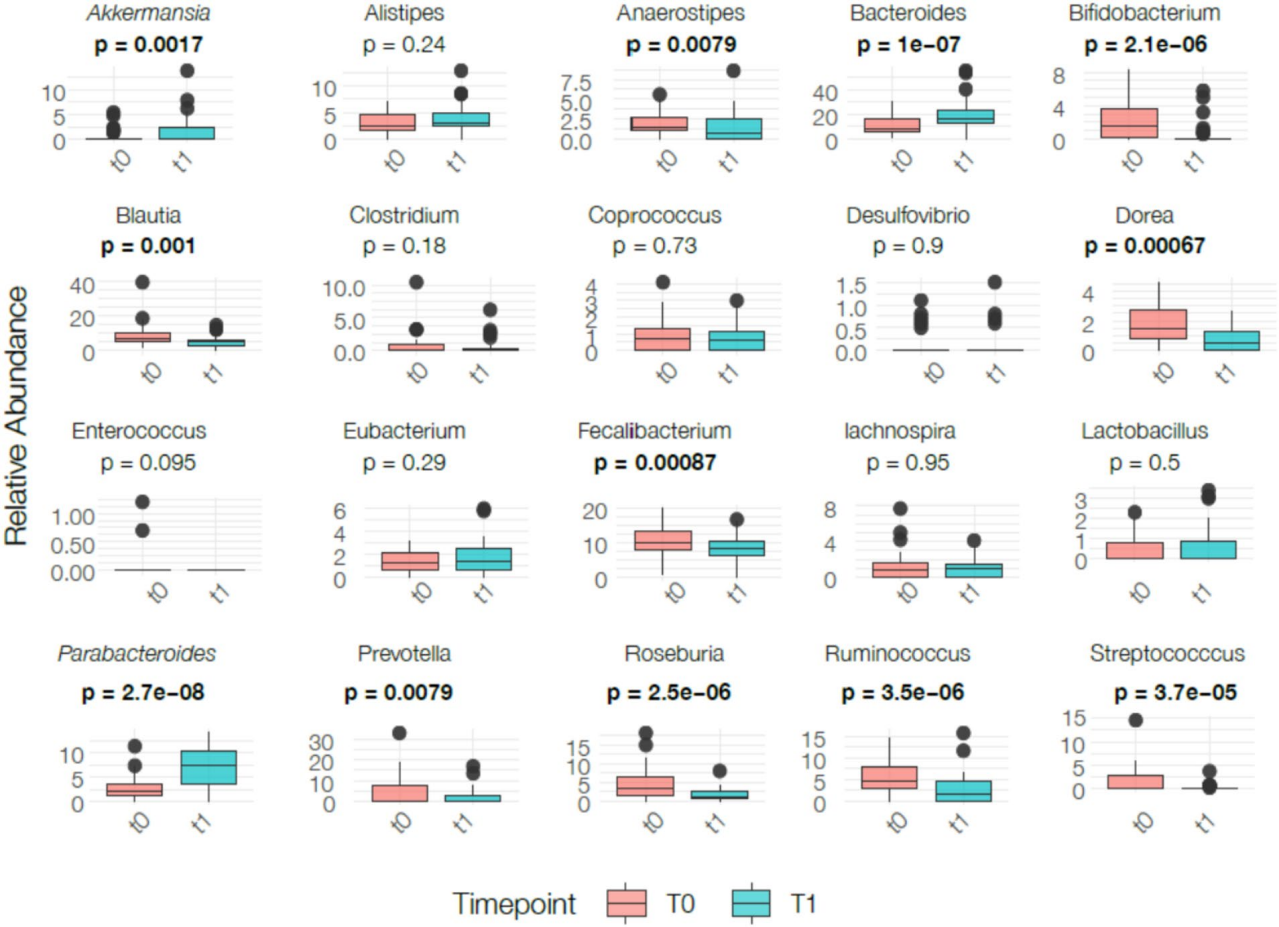
Genus-level relative abundance in the IBS subgroup at baseline (T0) and post-intervention (T1). Box plots show normalized abundance values (total-sum scaling), including individual variability, median, IQR, and range. Statistical comparisons were performed using paired *t*-test or Wilcoxon signed-rank test as appropriate. Significant results are indicated with bold *p*-values.

Moreover, a significant increase in *Akkermansia* (*p* < 0.05), *Bacteroides* (*p* < 0.0001), and *Parabacteroides* (*p* < 0.0001) was observed. Conversely, the abundance of several genera significantly decreased, including *Anaerostipes* (*p* < 0.05), *Bifidobacterium* (*p* < 0.0001), *Blautia* (*p* = 0.001), *Dorea* (*p* < 0.05), *E. coli* (*p* < 0.05), *Faecalibacterium* (*p* < 0.05), *Prevotella* (*p* < 0.05), *Roseburia* (*p* < 0.0001), *Ruminococcus* (*p* < 0.0001), and *Streptococcus* (*p* < 0.0001) (Figure 8). As in the whole population, the reduction of genera typically considered beneficial (e.g., *Bifidobacterium, Blautia, Faecalibacterium, Roseburia*) should be interpreted with caution and cannot be considered clinically adverse or favorable in the absence of inflammatory or biochemical markers. These variations may reflect changes in substrate availability under caloric restriction rather than IBS-specific responses.

**Figure 9:**
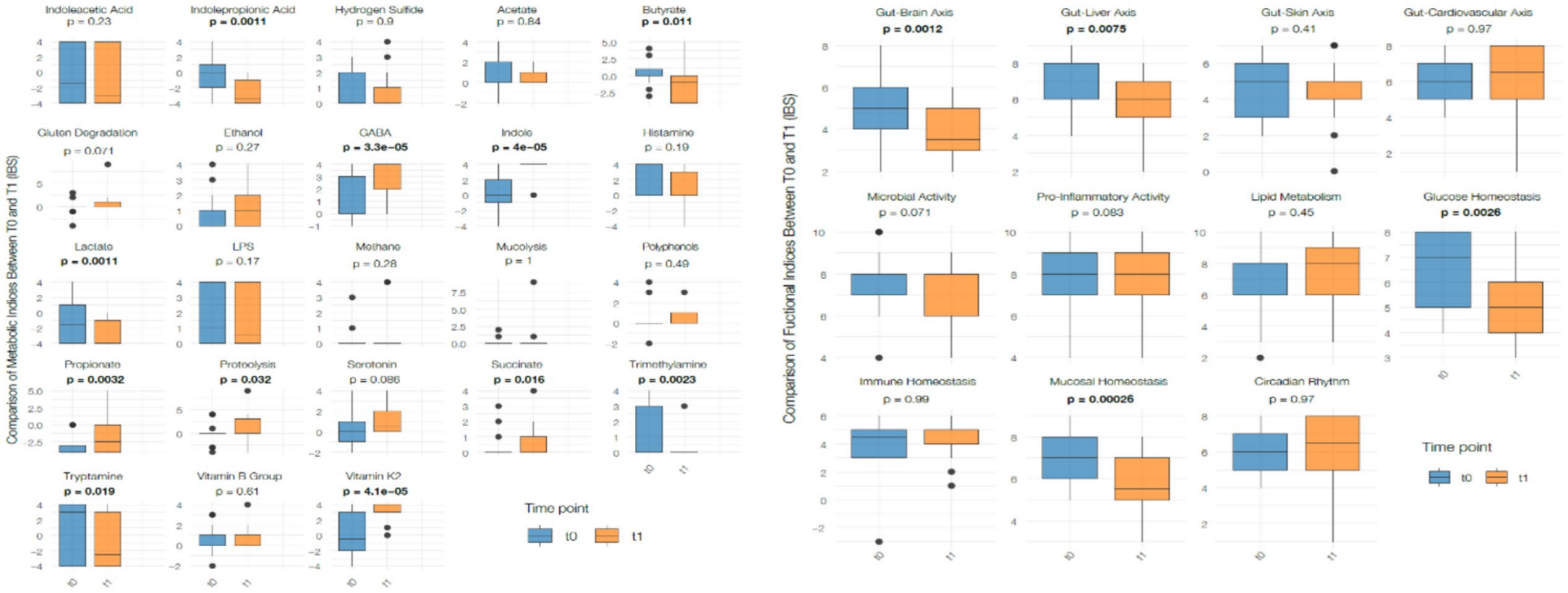
Changes in metabolic (left panel) and functional (right panel) indices between baseline (T0) and post-intervention (T1) in the IBS subgroup. Box plots display normalized index values (total-sum scaling), depicting median, IQR, range and individual observations. Statistical analyses were based on paired *t*-test or Wilcoxon signed-rank test according to normality. Significant differences are reported using bold *p*-values.

Regarding metabolic indexes, similar to the whole population, the IBS subgroup exhibited a significant reduction in the production of butyrate (*p* < 0.05), lactate (*p* < 0.05), indolepropionic acid (*p* < 0.05), and tryptamine (*p* < 0.05). Conversely, the levels of GABA (*p* < 0.0001), indole (*p* < 0.0001), propionate (*p* < 0.05), trimethylamine (*p* < 0.05), vitamin K2 (*p* < 0.0001), succinate (*p* < 0.05), and proteolysis (*p* < 0.05) significantly increased. These metabolite values are derived solely from stool analysis and should be considered exploratory indicators of microbial metabolic output rather than systemic biochemical concentrations. Therefore, no physiological or clinical implications can be inferred. Moreover, among functional indexes, the IBS subgroup showed significant reductions in gut-brain axis (*p* < 0.05) and liver-gut axis scores (*p* < 0.05). Additionally, mucosal homeostasis (*p* < 0.05) and glucose homeostasis (*p* < 0.05) significantly decreased (Figure 9). Because these functional scores are computed using a proprietary algorithm based on relative taxonomic abundances, they are not validated clinical biomarkers and do not directly reflect host neuroendocrine, hepatic, or metabolic function. Given the small size of the IBS subgroup and the absence of systemic physiological measurements, these variations should be interpreted with caution and considered descriptive rather than indicative of clinically meaningful changes.

## 4 Discussion

The aim of this study was to evaluate the effect of a 28-day VLEKT on body composition, gut microbiota, and stool-derived microbial metabolites in subjects with excess weight. As expected for a hypocaloric intervention, significant reductions in body weight, BMI, and waist and hip circumferences were observed. Similar short-term weight loss effects have been reported in previous ketogenic or very-low-calorie diet studies (15).

Body composition analysis showed a proportional decrease in fat mass with a slight reduction in absolute lean mass (kg), resulting in a relative increase in fat-free mass percentage. Phase angle, BCM and hydration parameters remained stable, suggesting preserved cellular integrity despite caloric restriction—findings consistent with other VLCKD-based interventions (19). These results indicate that VLEKT improved body composition without affecting markers of cellular function.

Food diary data confirmed a marked reduction in carbohydrate intake and a parallel decrease in lipid intake. The observed increase in total fiber intake was mainly related to the added soluble and insoluble fibers contained in meal-replacement products. Although some IBS participants reported reduced abdominal bloating, these symptom variations should be interpreted cautiously due to the lack of objective inflammatory or permeability markers. Thus, gastrointestinal tolerability appeared acceptable, but no mechanistic conclusions can be drawn.

No significant modifications were observed in dysbiosis or biodiversity indexes. The microbial changes detected—such as decreased Firmicutes and increased Bacteroidetes and Verrucomicrobia—mirror patterns frequently described during caloric restriction and nitrogen-shifted diets (20, 21). These trends likely reflect reduced availability of fermentable carbohydrates rather than specific effects of ketosis.

The rise in *Akkermansia* has also been repeatedly associated with weight-loss interventions and reduced energy intake (22). This increase aligns with previous findings, although the absence of inflammatory or metabolic biomarkers prevents further interpretation.

Importantly, short-term dietary interventions are known to rapidly shift gut microbiota composition, even within days, independently of macronutrient profile (23). Therefore, in the absence of a control group, natural temporal variability cannot be excluded as a contributing factor. For this reason, microbiota changes should be considered descriptive rather than mechanistic.

Overall, the microbial and metabolite changes observed in this study should be considered secondary adaptations to energy restriction rather than determinants of the anthropometric outcomes. This interpretation is in line with current literature showing that caloric deficit is the dominant driver of both weight loss and associated microbiota remodeling (24). Thus, the microbiota findings contextualize the anthropometric changes rather than explain them.

### 4.1 Gut microbiota analysis

Fecal sample analysis revealed a significant decrease in Firmicutes in both the total population and the IBS subgroup, consistent with evidence linking higher Firmicutes abundance to obesity and increased energy harvest (25). The Firmicutes phylum is functionally diverse: some genera such as *Streptococcus* and specific *Ruminococcus* species are associated with increased energy extraction and gastrointestinal symptoms, whereas others such as *Roseburia* and *Lactobacillus* contribute to short-chain fatty acid (SCFA) production and intestinal barrier support (26). This diversity makes phylum-level interpretation complex and requires caution.

In this study, reductions in *Ruminococcus* and *Roseburia*—both involved in fiber fermentation—were observed. While *Roseburia* is known for its butyrate-producing activity and its role in promoting epithelial health (27), no inflammatory markers were collected, therefore, no causal inference regarding symptom improvement is possible. Similarly, *Lactobacillus* and *Bifidobacterium* contribute to lactic acid production, pH regulation, and colonization resistance (28), yet their reduction must be interpreted cautiously.

A decrease in *Blautia*, another taxon associated with anti-inflammatory activity and metabolic benefits (29), and in *Faecalibacterium*, a key SCFA producer crucial for mucosal integrity (30), suggests that some microbial shifts could represent potentially unfavorable changes. However, without biomarkers of inflammation or permeability, the clinical relevance of these changes cannot be assessed.

The increase in Bacteroidetes and Verrucomicrobia observed between T0 and T1 aligns with known effects of caloric restriction and protein-rich diets (21, 23). The increase in *Bacteroides*, an important SCFA producer, is consistent with dietary shifts toward higher protein availability, while increases in *Parabacteroides*—linked to improved metabolic profiles and bile acid modulation—have also been described in weight-loss studies (31). These observations collectively suggest microbial restructuring driven by nutrient availability rather than by VLEKT-specific mechanisms.

*Prevotella* exhibited a reduction, a finding that is difficult to interpret given conflicting evidence: some studies associate *Prevotella* with inflammation and IBS symptoms (32), while others report improved glucose homeostasis with *Prevotella*-rich diets (32). Importantly, in this study no worsening of symptoms or inflammation-related findings were observed. Thus, changes in *Prevotella* remain context-dependent and inconclusive.

A reduction in Actinobacteria, particularly *Bifidobacterium*, was also detected. While *Bifidobacterium* is considered beneficial and associated with metabolic and immune health (33), its decline during short-term dietary restriction is reported in other hypocaloric interventions.

Finally, Verrucomicrobia—primarily represented by *Akkermansia*—showed a positive trend. *Akkermansia muciniphila* has been associated with improved metabolic health and enhanced mucosal barrier integrity in obesity (22). However, without biochemical markers, this finding remains descriptive.

Proteobacteria did not change significantly. Since high Proteobacteria levels (>8%) are considered a marker of dysbiosis (34), their stability suggests no major shifts toward dysbiotic patterns.

It is important to emphasize that the weight and body composition changes observed in this study are primarily attributable to the hypocaloric nature of the intervention, and not to changes in microbiota composition, which should be interpreted as secondary, correlational responses to dietary restriction.

Overall, the microbiota profile shifted toward taxa favoring protein and lipid degradation over carbohydrate fermentation, a pattern consistent with caloric restriction and reduced carbohydrate availability (24). In the absence of systemic biomarkers or a control group, the physiological significance of these microbial shifts remains speculative.

### 4.2 Metabolomic analysis

Metabolomic analysis revealed a reduction in butyrate, a key SCFA for intestinal health, in both the total population and the IBS subgroup. This finding is consistent with the observed decrease in several Firmicutes genera—major butyrate producers such as *Faecalibacterium* and *Roseburia*—which often decline during short-term caloric restriction (26). This reduction likely reflects decreased fermentation of dietary fibers rather than adverse physiological processes.

Conversely, propionate increased in parallel with the rise in Bacteroidetes, which are primary propionate producers (35). Although propionate has been associated with appetite regulation and glucose metabolism, its physiological effects are highly context-dependent, and no blood biomarkers were collected in this study; therefore, any mechanistic interpretation remains speculative. These metabolite changes should be viewed as markers of microbial metabolic output rather than systemic metabolic status.

Glucose restriction also led to a decrease in lactate and an increase in GABA, a metabolite produced by several gut bacteria and involved in gut–brain communication (36). While reductions in tryptophan-derived indolepropionic acid were detected—particularly in the IBS subgroup— this metabolite’s anti-inflammatory properties are described mainly in systemic circulation rather than stool (37). No significant differences in serotonin were observed, likely due to the reduced intake of tryptophan-rich foods.

Vitamin K2 increased in both groups, a finding compatible with changes in dietary substrates and microbial taxa involved in menaquinone biosynthesis. However, without biochemical data, the hypothesis of altered biliary metabolism cannot be confirmed. Overall, these metabolite variations should be considered descriptive indicators of microbial metabolic output rather than systemic metabolic alterations.

Functional indices showed reductions in gut–brain and liver–gut axis scores, as well as in mucosal and glucose homeostasis indexes. As these indices are computed using a proprietary algorithm based solely on taxonomic composition, they should not be interpreted as measures of host physiological function. Short-term dietary interventions are known to transiently alter microbial metabolism and epithelial interactions (24). However, without inflammatory, hormonal, or permeability biomarkers, the physiological significance of these changes cannot be assessed.

Taken together, the metabolomic and functional index findings remain exploratory. They describe microbiota-related metabolic patterns observed in stool but cannot be extrapolated to systemic health outcomes. In the absence of biochemical confirmation of ketosis or systemic metabolic markers, these results should be interpreted as descriptive signals requiring further investigation.

### 4.3 Limitations

This study has several limitations. The sample size was small, and the single-arm, uncontrolled design limits both statistical power and generalizability. The 28-day duration and absence of long-term follow-up do not allow assessment of sustained effects. No inflammatory, metabolic, hormonal, or permeability biomarkers were collected. Dietary intake was self-reported and therefore subject to recall and reporting bias. Biochemical confirmation of ketosis (urine or blood ketones) was not performed; adherence and macronutrient intake were used as proxies for ketosis induction. Because no biochemical markers of ketosis were collected, the presence and intensity of nutritional ketosis could not be objectively verified and should be considered a limitation of the study.

Microbiota-derived metabolic and functional indices were generated using a commercial algorithm and represent exploratory, non-validated stool-based estimates that do not reflect systemic physiology. In addition, natural temporal variability in gut microbiota composition cannot be excluded in the absence of a control group. Another limitation is that body composition was assessed using bioelectrical impedance analysis (BIA) rather than gold-standard methods such as dual-energy X-ray absorptiometry (DEXA) or nuclear magnetic resonance (NMR). Furthermore, the observed increase in fat-free mass (FFM) percentage should be interpreted cautiously, as it resulted from a proportionally greater reduction in fat mass rather than an actual increase in absolute FFM, which showed a slight decrease. Finally, because multiple statistical comparisons were performed without correction, the risk of type I error is increased. Overall, the observed changes in body composition, microbiota, and metabolites should be interpreted as descriptive and hypothesis-generating rather than conclusive evidence of clinical benefit.

## 5 Conclusion

The findings confirm that VLEKT is effective in promoting short-term weight loss and favorable changes in body composition, with notable reductions in body weight, BMI and abdominal adiposity.

Microbiota and metabolite analyses revealed several shifts commonly associated with energy restriction and macronutrient redistribution. Increases in taxa such as *Akkermansia* and *Bacteroides*, along with changes in microbial metabolites including propionate, GABA and vitamin K2, reflect alterations in microbial metabolic activity during the intervention. However, these stool-derived measurements do not directly reflect systemic physiology, and their clinical significance remains uncertain.

Overall, the changes observed over 28 days suggest that VLEKT can rapidly influence both anthropometric parameters and gut microbial patterns. Nevertheless, the short intervention duration, lack of a control group and absence of biochemical markers of inflammation, permeability or ketosis limit the interpretability of these findings. VLEKT remains a selective nutritional intervention that should be followed under professional supervision. Further controlled and longer-term studies are required to assess the durability of these effects and clarify their broader implications for metabolic and gastrointestinal health.

## Data Availability

The datasets presented in this article are not readily available because it contains information that could compromise the privacy of research participants. Requests to access these datasets should be directed to c.dirosa@unicampus.it.
